# Novel Modulators of Asthma and Allergy: Exosomes and MicroRNAs

**DOI:** 10.3389/fimmu.2017.00826

**Published:** 2017-07-21

**Authors:** Beatriz Sastre, José A. Cañas, José M. Rodrigo-Muñoz, Victoria del Pozo

**Affiliations:** ^1^Laboratory of Immunoallergy, Department of Immunology, IIS-Fundación Jiménez Díaz, Madrid, Spain; ^2^CIBER de Enfermedades Respiratorias (CIBERES), Madrid, Spain

**Keywords:** exosome, microRNA, asthma, allergy, inflammation, intercellular communication

## Abstract

Intercellular communication is crucial to the immune system response. In the recent years, the discovery of exosomes has changed the way immune response orchestration was understood. Exosomes are able to operate as independent units that act as mediators in both physiological and pathological conditions. These structures contain proteins, lipidic mediators, and nucleic acids and notoriously include microRNAs (miRNAs). miRNAs are short RNA sequences (around 19–22 nucleotides) with a high phylogenetic conservation and can partially or totally regulate multiple mRNAs, inhibiting protein synthesis. In respiratory diseases such as asthma and allergic sensitization, exosomes released by several cell types and their specific content perform crucial functions in the development and continuation of the pathogenic mechanisms. Released exosomes and miRNAs inside them have been found in different types of clinical samples, such as bronchoalveolar lavage fluids and sputum supernatants, providing new data about the environmental factors and mediators that participate in the inflammatory responses that lead to the exacerbation of asthma. In this review, we summarize our current knowledge of the role of exosomes and miRNAs in asthma and allergic sensitization, paying attention to the functions that both exosomes and miRNAs are described to perform through the literature. We review the effect of exosomes and miRNAs in cells implicated in asthma pathology and the genes and pathways that they modify in them, depicting how their behavior is altered in disease status. We also describe their possible repercussion in asthma diagnosis through their possible role as biomarkers. Therefore, both exosomes and miRNAs can be viewed as potential tools to be added to the arsenal of therapeutics to treat this disease.

## Introduction

Cellular communication is a key feature of the immune response. This communication is mainly done by cytokines, chemokines, and other soluble mediators. But in the past decade, extracellular vesicles have become recognized as players in these intercellular communications. There are a variety of these extracellular vesicles that have now been studied, and one of them are the exosomes, that contain a wide variety of molecules inside. One of them are microRNAs (miRNAs), important elements linked to regulation of functional cellular mechanisms that open a new door to genetic regulation (1). So, new almost unexplored areas of knowledge are emerging.

Exosomes are small vesicles (10–150 nm) released into the extracellular space after the fusion of multivesicular bodies (MVBs) with the cell membrane. They are secreted by several types of cells—among them, cells implicated in allergy and the immune response, including B and T lymphocytes, dendritic cells (DCs), mast cells (MCs), epithelial cells, and macrophage-monocyte cells (2–7)—and, as recently discovered by our group, by eosinophils (8). Also, other cell types, such as hematopoietic cells (reticulocytes), platelets, microglia, and some tumor cells, also produce exosomes (9–12). This etiologic heterogeneity led to find exosomes in a large variety of body fluids, including blood, amniotic fluid, urine, breast milk, and saliva, under physiological and pathological conditions (13, 14). Moreover, analysis of bronchoalveolar lavage fluid (BALF) and nasal lavage fluid documents that exosomes can be found on respiratory mucosal surfaces (15–17).

So, exosome secretion provides cells with a mechanism for rapid release of a selective repertoire of molecules, such as proteins, mRNA, lipids, and miRNAs. Once in the extracellular space, the exosomes can be taken up by neighboring cells or enter the circulation and travel to distant sites in the bloodstream, modulating the activity of receptor cells. Thus, exosomes have been implicated in a broad range of biological processes, such as immune regulation, inflammation, cancer progression and metastasis, neuronal communications, and lung diseases (18–22).

These structures commonly contain tetraspanins (CD63, CD81, and CD9), transmembrane receptors (integrins β1 and β2), membrane-trafficking proteins, adhesion molecules, lipid rafts, costimulatory molecules, proteins involved in MVBs formation, major histocompatibility complex (MHC) of class I and MHC of class II in the membrane, metabolic enzymes, chaperones, cytoskeletal proteins, signal transduction proteins, and nucleic acids. An important characteristic of exosomes is that their specific protein composition depends on their cellular or tissue origin and may differ according to the physiological or pathological situations that activate exosome release. Moreover, other components of exosomes include lipids (cholesterol, diglycerides, and phospholipids) and bioactive agents [prostaglandins and leukotrienes (LTs)] (23, 24). In addition, exosomes contain DNA and RNA molecules, including mRNAs and miRNAs, with important regulatory roles (25, 26).

Thus, exosomes are “professional transporters and messengers” to systemic level making them a possible tool for the therapeutic delivery of small interference RNAs (siRNAs), miRNAs and short hairpin RNAs (shRNAs) (27), or another kind of tools such as anti-inflammatory or/and anticancer agents (28–30).

In summary, exosomes are implicated in several processes related to immunoregulation (31), protective immune responses to infections (32), asthma (33), and allergy (3). The focus of this review is on the role of exosomes and miRNAs in the pathology of asthma and allergy.

Much of functional importance of exosomes lies to their role as “miRNAs containers.” miRNAs, are the best-known RNA subtype within a larger group of nucleic acids called “small RNAs,” an arbitrary term that was previously used for other non-coding RNAs such as small nuclear RNAs and transfer RNAs. The miRNAs are single-stranded RNAs with a length of approximately 22 nucleotides (mature miRNAs) generated from endogenous hairpin transcripts (34).

Currently, there are more than 2,500 entries for mature miRNAs in humans (35). A high degree of phylogenetic conservation has been observed between miRNAs of bilaterian animal, which implies that miRNAs have a crucial role in animal evolution (36). Mammalian miRNA genes have multiple isoforms (paralogs) that often have identical sequences at nucleotide positions 2–7 relative to the 5′ end that are called “seed sequence.” These six nucleotides are decisive in pairing with target mRNAs (37), conferring upon miRNAs one of their most important characteristics, namely promiscuity behavior. So, this feature implies that one miRNA can regulate multiple (several hundred) different mRNAs (38).

Some miRNAs are originated in independent transcription units (39) although approximately 50% of mammalian miRNA loci are in close proximity to other miRNAs. They can form clusters, and these can be interrelated, but this situation is not always bidirectional. So, they could be related between them but they do not form a cluster (40).

MicroRNAs regulate the expression of genes through two methods: translational repression and degradation of mRNA. These effects will depend on the degree of sequence complementarity between the miRNA 5′ region and the mRNA 3′-untranslated region (40–42). In animals, miRNAs bind to complementary sequences located in their target mRNAs, totally or partially inhibiting protein synthesis (43) and exerting an important effect at the translational level without modifying the mRNA load (44). A single miRNA can exert repression on multiple mRNAs. In this respect, mutation experiments have probed two classes of target mRNAs. One type of target exhibits a perfect Watson–Crick complementarity at the mRNA 5′ end. In this case, additional base pairing is not necessary. The other class has an imperfect 5′ end, but this is compensated by base pairing at the 3′ end of the miRNA (45). Thus, miRNAs are the regulators of protein synthesis at the post-transcriptional level and modulate gene expression by translational repression or degradation of a specific mRNA.

The most important trouble in miRNA study field is their target identification (46). Computational programs based on sequence alignment have been developed to perform predictions of miRNA targets. These predictions are more complex in animal RNAs, because complementary sequences are more imperfect than in plants. Computational tools are based on evolutionary-conserved regions and target-binding sites (47–49). The following two classes of bioinformatics platform exist: experimentally verified miRNA target databases and computationally predicted miRNA target databases.

So, exosomes and miRNAs are new players that could exert relevant effects in physiologic and pathologic mechanisms and that is not all, they could have an important role in new diagnostic and therapeutic approaches.

In the present review, we summarize the role known so far of exosomes and miRNAs in respiratory diseases, specifically, allergy and asthma pathology.

## Exosomes in Asthma and Allergy

Several cell types implicated in asthmatic and allergic processes can release exosomes that contribute to these pathologies. It is necessary to take into account the exosome load and composition and how this may depend on the cellular origins. So, both effector and structural cells implicated in asthmatic pathophysiology contribute to exosome pool that acts in inflammatory context that characterize this disease.

### Exosomes from DCs and Monocyte-Macrophages

Dendritic cells are a specialized cell type of mammalian immune system. They mediate innate immunity and they are able to phagocyte pathogens, but their principal function is to process and to present antigens to T-lymphocytic cells. They are antigen-presenting cells (APCs) with the essential properties required for the effective induction of an immune response (50). Several studies have demonstrated that these cells are able to secrete exosomes; and like their parent cells, these exosomes contain MHC molecules of both classes (I and II), and they are able to specifically stimulate T-cell responses, using indirect interaction mechanisms (51). DC-derived exosomes develop these responses by direct contact with CD4^+^-T cells or *via* the capture of these exosomes by other APCs (51, 52).

Dendritic cell-derived exosomes can carry aeroallergens and contribute to allergic inflammation, as previously described by Vallhov et al. (3). These nanovesicles are able to present allergens and to induce the production of T helper (Th) 2 cytokines in T cells in allergic subjects. These exosomes can to induce interleukin (IL)-4 responses in peripheral blood mononuclear cells (PBMCs) from cat-allergic donors and not in feline-antigen tolerant donors, when exosomes present the antigen Fel d 1. These findings may be important for future exosomes engineering to create exosome-based vaccines for immunotherapy.

Previous studies have characterized the surface protein components of exosomes from DCs. Admyre et al. (53) determined that monocyte-derived DC exosomes express several molecules such as HLA-DR, MHC class I, CD63, CD86, and CD54. The presence of CD63 confirmed the endosomal origin of these vesicles, and CD86, which is a costimulatory molecule, showed the implication in clonal expansion and differentiation of T cells. Through CD54 (an adhesion molecule), exosomes can interact with lymphocyte function-associated antigen (LFA)-1 present in T cells.

In contrast, the release of monocyte-derived macrophage (MDM)-derived exosomes is modulated by several factors. Regulatory cytokine transforming growth factor beta 1 (TGF-β1) affects exosome formation to decrease the rate of exosome delivery (7). Also, the Rab guanosine triphosphate phosphatases serve as master regulators of membrane trafficking and they are involved in the exosome release mechanism (54). In contrast, cytokines such as IL-1β induce microvesicular shedding from peripheral blood monocytes (55) and interferon-gamma (IFN-γ) produces an increase in exosome secretion by alveolar macrophages (6). So, it has been hypothesized that lung macrophages might be a source of exosomes implicated in Th1 but not Th2 inflammation; although this possibility must be confirmed.

Besides exosome antigen-presenting capacity, MDM- and DCs-derived exosomes contain a functional enzyme activity *in vitro* that has a potential role in inflammation. Esser et al. (7) demonstrated that the enzymatic capacity of DCs-derived exosomes can convert LTs A_4_ to other LTs (LTB_4_ and LTC_4_). Also, they demonstrated that these exosomes induce polymorphonuclear leukocyte migration and granulocyte recruitment to inflammatory sites. Both 5-keto eicosatetraenoic acid (KETE) and LTB_4_ are potent chemoattractant agents for eosinophils and neutrophils. These molecules are arachidonic acid metabolites produced by MDM-derived exosome enzymatic activity. LTs are pro-inflammatory lipid mediators, which play key roles in the pathogenesis of asthma, allergy, and chronic inflammation.

### Exosome from MC- and Basophil-Derived Granules

Mast cells are granulated cells located in all vascularized tissues, near blood vessels, smooth muscle cells, mucous glands, and hair follicles. They originate from hematopoietic cells and present a large number of granules which primarily store histamine and heparin (56). MCs are key effector cells in Th2- and IgE-associated immune responses and their activation contributes to resistance to parasites and can drive allergic reactions, such as those involved in anaphylaxis and asthma (57).

It has been reported that MCs from both humans and mice can release exosomes (58). The composition of these exosomes is quite heterogeneous and includes factors such as CD13, ribosomal protein 6 kinase, annexin V, Cdc25, phospholipases, heat shock proteins (HSPs), immunological factors (MHC class II), costimulatory factors (CD86, CD40, and CD40L), and adhesion molecules including LFA-1, intercellular adhesion molecules-1 and mRNAs, small RNAs, and miRNAs (56).

Mast cell-derived exosomes can interact with different cell types through immunoregulatory proteins as well as mRNAs and miRNAs (59). Exosomes from MCs provide cell contact signals through CD40 ligands and can stimulate purified B cells to produce IgE through a T-cell independent mechanism (60). Moreover, these exosomes can induce lymphocyte stimulation and proliferation, leading to the production of IL-2, IL-12, and IFN-γ (but not IL-4), and Th1 responses (61). Moreover, these exosomes induce the production of pro-inflammatory cytokines such as IL-6 and IL-8 that are released from human airway smooth muscle cells (ASMCs), exacerbating airway inflammation and recruiting inflammatory cells that perpetuate asthmatic symptoms (5). Finally, MC-derived exosomes induce phenotypic functional maturation of DCs *in vivo* and *in vitro*, improving the immune response (62).

Similar to MCs, basophils are granulated leukocytes derived from granulocyte-monocyte progenitor cells though they exist in a mature state (63). They are present in the circulation in low numbers; however, in a infection and/or inflammation situation, they increase in number and migrate from the bloodstream to infected or inflamed tissue sites. To our knowledge, there are no data related to exosome production by basophils. However, Dvorak (64) reported that these cells contain numerous granules that often have contents resembling exosomes and may be associated with a form of mediator exocytosis.

### Eosinophil-Derived Exosomes

Eosinophils are multifunctional granulocytes implicated in the pathogenesis of inflammatory processes in asthma and helminthic infections. They have been considered to be end-stage cells involved in host defenses against parasites. Eosinophils play a central role in asthma pathogenesis, releasing an array of pro-inflammatory cytokines (IL-2, IL-4, IL-5, IL-10, IL-12, IL-13, IL-16, IL-18, and TGF-α/β), chemokines (RANTES and eotaxin-1), and lipid mediators (LTC_4_, platelet-activating factor, thromboxane B2, and prostaglandins) that orchestrate the processes and symptoms of the disease (65, 66). These cells carry a large number of granules in their cytoplasm that contain cytotoxic granule proteins, including major basic protein (MBP), eosinophil cationic protein (ECP), eosinophil-derived neurotoxin (EDN), and eosinophil peroxidase (EPO) (65, 66). In addition, several studies have reported that eosinophils can act as APCs, with phagocytic capacity as well as MHC class II and costimulatory molecule expression (67, 68).

Although eosinophils are important for allergic and asthmatic processes, no previous studies have been conducted to remark some important aspect of their physiology for asthma and allergy like exosome secretion. To date, there are only two published studies by our group that have characterized eosinophil exosomes and their implications for the pathogenesis of asthma (8, 69). Eosinophils secrete nanovesicles that are consistent in size (162 nm diameter, by NanoSight measurement), shape (cup-shape morphology), and composition (presence of CD63, CD9, and ALIX) with exosomes. CD63, CD9, and ALIX proteins indicate that these eosinophil nanovesicles have an endosomal origin from MVBs. Moreover, these exosomes carry eosinophil characteristic proteins, such as MBP and EPO, which could produce tissue damage and asthma exacerbations. Also, exosome secretion is significantly higher in eosinophils from asthmatics than in healthy donors (8).

Regarding their functional effects on eosinophils, we have reported that exosomes from the eosinophils of asthmatic patients induce in these cells a rise in the production of reactive oxygen species. Also, these exosomes increase the migratory capacity in eosinophils, as well as, their adhesion and adhesion molecule expression, which are important processes in asthma. By contrast, eosinophil-derived exosomes from healthy patients do not affect to asthmatic eosinophil functionality (69). Also, we have described the exosome uptake mechanism of eosinophils which involves direct internalization of the whole vesicle, corresponding with one of the two previously described uptake mechanisms (70). Finally, we provided a list of the eosinophil-derived exosome proteins from a pool of both asthmatic patients and healthy volunteers and confirmed the presence of proteins implicated in different cell functions, such as (A) classical eosinophil proteins (MBP, ECP, EDN, and EPO), (B) S100 proteins (S100A8 and S100A9) implicated in antifungal and antibacterial activity, promotion of cytokine and chemokine production, induction of pro-inflammatory responses in monocytes, and chemotactic factors for immune cells (17), (C) HSP with a molecular weight of 70 kDa, implicated in protein folding, (D) several integrins (integrin α_M_, β_2_, and β_3_) related to cell adhesion, (E) periostin and filaggrin, important proteins implicated in asthma pathogenesis, and (F) metabolic enzymes (α-enolase, catalase, and arachidonate 15-lipoxygenase).

### Exosomes Released by Lymphocytes

Lymphocytes have important actions in the immune response. In asthma and allergy, B lymphocytes produce and release IgE against specific allergens or antigens, leading to the degranulation of basophils and MCs, and histamine release. T lymphocytes mediate cytokine changes and shifts to the Th2 phenotype characteristic of asthmatic pathology. Moreover, these cells secrete type 2 cytokines that trigger a series of events including granulocyte migration as well as cytokine and chemokine release by other cell types that drive pro-inflammatory effects and development of the characteristic features of asthma.

Several studies have demonstrated that T lymphocytes can release exosomes (71, 72) and that CD8^+^ T cells can secrete exosomes with cytolytic properties (4). However, no functional studies have been carried out in asthma or allergy documenting release of exosomes from T cells. By contrast, investigations have shown that exosomes from other cell types can stimulate T cells and have effects on asthma and allergic responses. Hough et al. (31) compiled several studies of exosome function for immunoregulation in chronic diseases, including asthma. The authors show that exosomes of different origins (B cells, DCs, and BALF) can stimulate activation, proliferation, and Th2 cytokine production by CD4^+^ T cells in different ways.

In contrast, a larger number of studies of B-cell-derived exosomes have been presented. After Raposo et al. (52) reported that exosomes carry MHC class II receptors and specifically present antigenic peptides to T cells, other authors performed further studies of this process. All have concluded that B-cell-derived exosomes present MHC class II, costimulatory molecules (CD40, CD80, and CD86) and integrins (β1 and β2) and can induce T-cell responses (73–75). Also, HSPs in the B-cell exosomes are important to the maturation of DCs (76). Admyre et al. (2) published a study related to B-cell-derived exosomes affected T-cell proliferation and Th2 cytokine production. In the study, it was found that the amount of birch peptide (Bet v 1) in exosomes from B cells needed for induction of proliferation on T cell. Moreover, this exosome-associated peptide induced a Th2 cytokine response resembling that observed in stimulation directly with B cells, characterized by the release of high levels of IL-4, IL-5, and IL-13 and low levels of IFN-γ, and tumor necrosis factor (TNF)-α. These results indicated an important role for exosomes in allergic responses by acting as independent “antigen-presenting structures” for T cells without direct cell-to-cell contact. In contrast, exosomes from B cells and other cell types, present in serum or BALF, have been described like allergic tolerogenic particles called tolerosomes (77, 78).

### Exosomes from Structural Lung Cells in Asthmatic and Allergic Processes

Several types of anatomical changes are implicated in the characteristic processes involved in asthma progression: airway remodeling, bronchoconstriction, and mucus hypersecretion. The key cellular changes involve ASMCs, goblet cells, and epithelial cells (79). There are no studies addressing exosomes released by ASMCs or goblet cells; however, there are a few studies reporting the effect of exosomes from BALF or produced by other cell types on airway muscle cells.

The ASMCs are important elements in bronchoconstriction and airway remodeling. During asthma progression, hyperplasia and hypertrophy of the airway tissues occur and pro-inflammatory cytokines are released from the structural airway cells.

There are some studies of exosomes acting over airway muscle cells. Xia et al. (5) showed that MCs can stimulate human ASMCs by indirect contact. MCs can adhere to ASMCs *via* cell adhesion molecule 1 and induce the release of cytokines and chemokine generation by the ASMCs, leading to the recruitment of more MCs, perpetuating the asthma symptoms. This stimulation can occur *via* exosomes, and depletion of MC-derived exosomes can contribute to reduction of the stimulatory response, although depletion of other soluble factors is also an important factor in this effect. Recently, it has been reported that lipopolysaccharide-stimulated neutrophil-derived exosomes affect horse airway smooth muscle remodeling (80). The study shows how horse ASMCs internalized the exosomes from stimulated neutrophils to their cytoplasm and how these exosomes then modulated apoptosis and induced proliferation of the ASMCs, thus contributing to the process of airway remodeling.

The airway epithelium is the primary barrier against external irritants and toxins, such as air pollutants, irritants, allergens, and pathogens. Airway epithelial cells (AECs) constitute the surface layer of the bronchi and alveoli, facing the lumen, and they are in direct contact with the external environment. These cells actively contribute to the establishment and progression of asthma and allergic airway inflammation by orchestrating the inflammatory response to exogenous stimuli, such as allergens and cigarette smoke (6).

Exosomes have been described from different types of AECs. The exosomes show different sizes and shapes, and it has been concluded that their morphology depends on the cell type, and whether there is apical or basal release of the organelles (81). It has also been concluded that these exosomes carry important molecules (mucins and sialic acid) that have been implicated in innate defense mechanisms and that can modulate airway inflammation (16).

Kulshreshtha et al. (6) confirmed the effect of exosomes from bronchial epithelial cells (BECs) on inflammatory processes. Th2 cytokines, principally IL-13, increased the secretion of exosomes from stimulated BECs, and these exosomes could induce monocyte proliferation. Moreover, the cytokine IL-13 modified the composition of these exosomes. Beyond cytokines, other factors such as mechanical stress can stimulate exosome secretion. During episodes of bronchoconstriction in asthmatics, compressive mechanical stress occurs that induces an increased exosome secretion from BECs (79). The effect of BALF-derived exosomes on AECs has been studied by Torregrosa Paredes et al. (15). They reported that exosomes from BALF can stimulate AECs and contribute to inflammation by increasing cytokine (IL-8) and LT (LTC_4_) generation.

**Figure 1:**
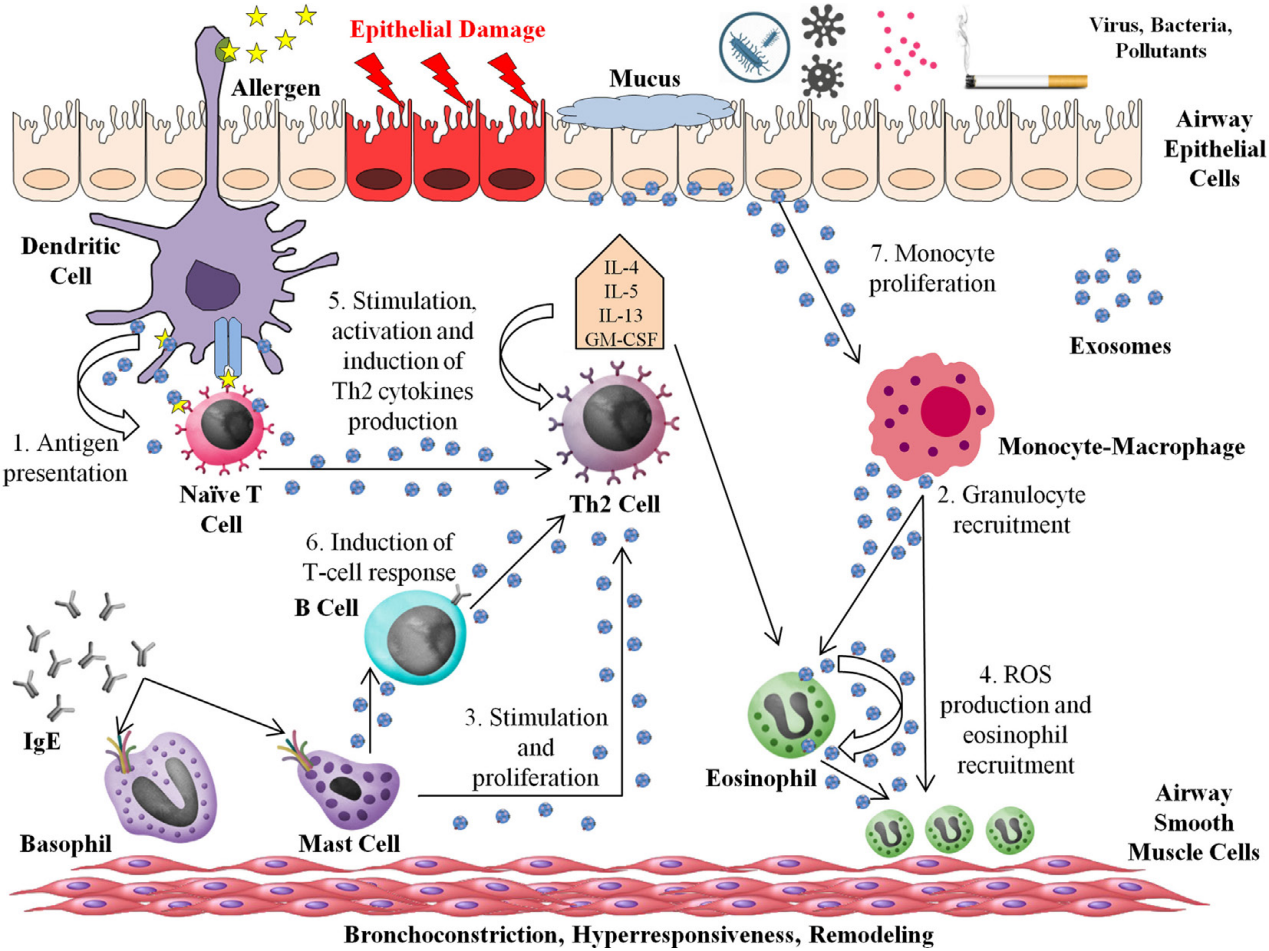
Roles of exosomes in asthma and allergic processes. The entry of the allergen into the airway triggers the Th2 response. Mature dendritic cells (DCs) induce the differentiation of naive CD4^+^ T cells into CD4^+^ Th2 cells. The majority of immune cells are able to produce and release exosomes to the Th2 environment, acting in several ways: (1) DC-derived exosomes can stimulate T-cell responses and act as an “antigen-presenting unit.” (2) Macrophage-derived exosomes contain functional enzymes that play a potential role in inflammation, synthesizing leukotrienes (LTs) and recruiting granulocytes to the inflammation site. (3) Exosomes from mast cells can stimulate B cells and induce simulation and proliferation of others lymphocytes. (4) Eosinophil-derived exosomes increase the pro-inflammatory capacity of the same or other eosinophils, producing higher quantities of reactive oxygen species and increasing the migration of other eosinophils to the inflammation site. (5) T cells produce exosomes that are able to stimulate, activate, and increase Th2 cytokine release. (6) B-cell-derived exosomes present major histocompatibility complex class II and costimulatory molecules, which are able to induce T-cell responses. (7) Airway epithelial cell-derived exosome carry a range of different molecules implicated in the modulation of inflammation, inducing monocyte proliferation and contributing to increasing cytokine and LT generation. The secretion of exosomes from airway epithelial cells is increased by Th2 cytokines as interleukin-13.

Figure 1 depicts a simplified model of the role of exosomes in the interaction between different immune cells in allergic reactions and asthma.

## miRNAs and Asthma

Asthma is a heterogeneous disease; therefore, traditional diagnostic approaches have utilized a classification system in which patients are divided into subgroups according to etiologic diagnosis and treatment. For this purpose, the term endotype was created to distinguish asthmatics not only by their clinical characteristics (phenotype) but also by the pathophysiological characteristics of their disease (82). Currently, to generate new classifications of asthmatic patients, biomarkers of asthma need to be discovered.

The characterization of miRNA expression and its role in asthma has been developed using different approaches. So, miRNA expression in different tissues and cells that are implicated in the pathogenesis of asthma has been studied to determine differential expression between healthy patients and those with active asthmatic disease status. miRNAs that were found to be deregulated in asthmatic samples have been further studied in asthma models, both *in vitro* and *in vivo*, to elucidate their functions in asthmatic disease.

**Figure 2:**
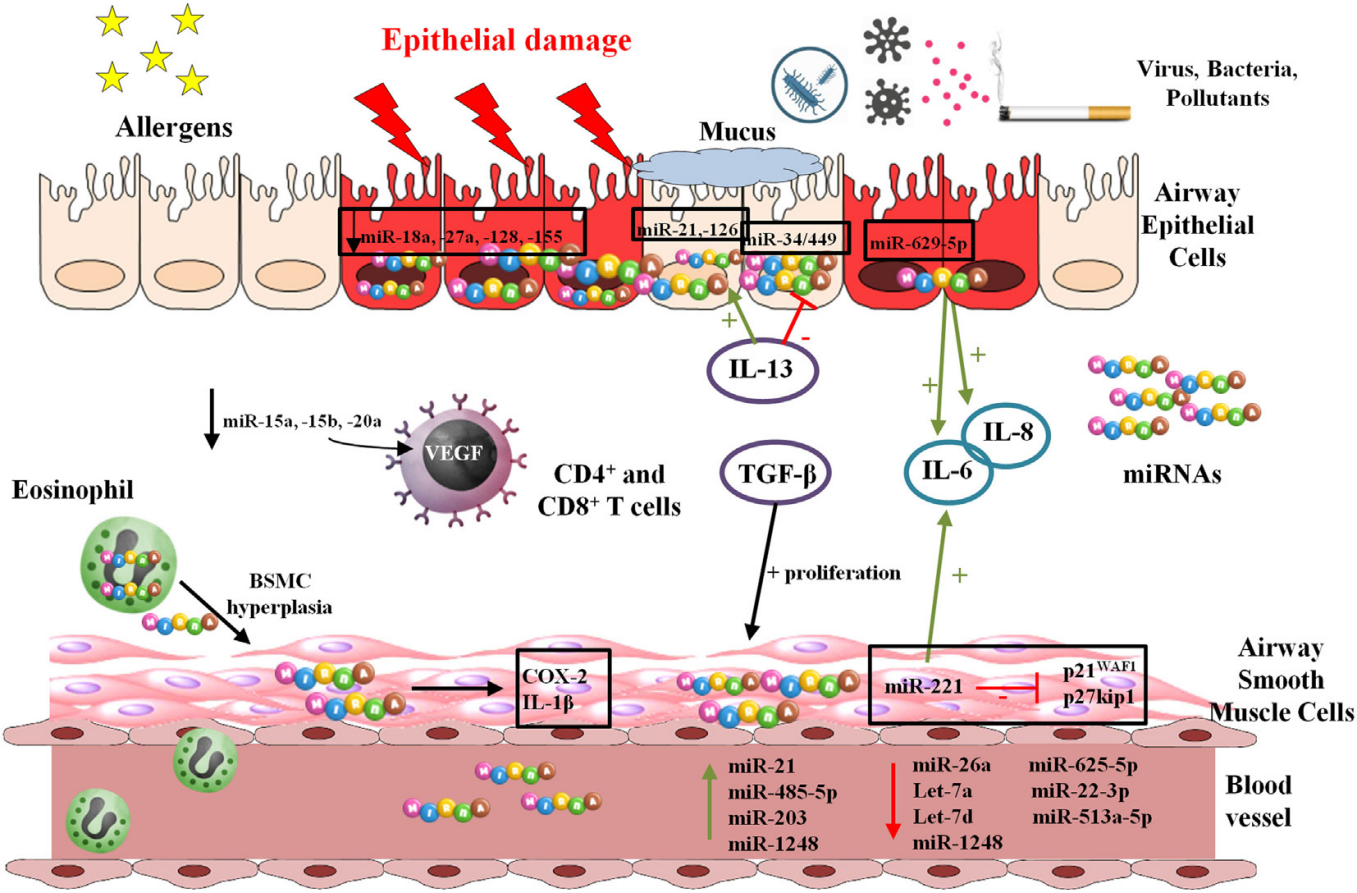
Functions and mechanisms involving microRNAs (miRNAs) in asthma and allergy. miRNAs are preprocessed in the cellular nucleus and completion of processing and maturation occurs in cytoplasm. They are usually delivered inside exosomes by multiple cell types exerting their effects on the own producing cells or on other cellular types.

Figure 2 shows possible targets of miRNAs in asthma pathogenesis.

### miRNAs Expression in Structural Cells: Airway Biopsies, BECs from Epithelial Brushings and Airway Muscle Cells

Most of the studies of miRNAs in asthma are characterized by being quite heterogeneous. Within the structural airway cells, several cell lines and samples have been studied like nasal biopsies, epithelial cells with different origins or airway muscle cells.

Some studies have employed airway biopsies from asthmatic patients to analyze miRNA expression in whole tissue. Suojalehto et al. (83, 84) carried out several studies using nasal biopsies from different populations (allergic and non-allergic rhinitis patients and long-term asthmatic patients) to evaluate miRNAs expression. Study focused on rhinitis pathology (83) showed a higher expression of miR-498, miR-155, and miR-205 in allergic rhinitis population in comparison to control and non-allergic rhinitis subjects. Also, let-7 expression was downregulated in the allergic rhinitis plus asthma group compared to controls and the non-allergic rhinitis group (83). However, some of these upregulated miRNAs were found downregulated in a long-term asthmatic subjects compared to controls (miR-155 and let-7), although miR-498 showed a similar pattern. Also, in the case of long-term asthmatic patients, miR-155 (an upregulated miRNA in rhinitis study) showed a positive correlation with fractional exhaled nitric oxide levels, nasal nitric oxide levels, and IL-13 mRNA levels (84), being a proof of heterogeneous performance of miRNAs.

Using airway biopsies from controls and mild asthmatics before and after corticosteroid therapy, another study showed no differences in miRNA expression patterns by quantitative PCR (qPCR) between these groups, in contrast to previous results. This could be due to the mild asthmatic phenotype. They also observed cell-specific miRNA expression profiles from lung biopsy, demonstrating elevated heterogeneity in these tissue biopsies (85).

Full tissue biopsies are conformed by high cellular heterogeneity, making gene expression profiling difficult to achieve in a cell-specific manner. To address this problem, bronchial epithelial brushings have been employed to analyze miRNAs expression in a single cell type. BECs play important roles in asthma, producing mucus and participating in airway remodeling (86). So, different research groups have focused their attention on this type of cellular population as reference sample in the study of asthma. Solberg et al. (86) showed a differential expression of several miRNAs, including downregulation of the miR-34/449 family, in bronchial epithelial brushings from controls, steroid naive asthmatics (before and after inhaled corticosteroids), and steroid-treated asthmatics, being IL-13 a repressor of this miRNA family (*in vitro* assays) not affecting their expression the corticosteroid treatment.

Different groups have confirmed the role of some cytokines, such as IL-13, in the modulation of miRNAs. In this way, Wu et al. (87) observed an effect of IL-13 on the expression of miR-21 and miR-126 that were upregulated in BECs from asthmatics (with or without inhaled corticosteroids treatment). So, when the BECs were cultured with IL-13, a positive correlation between miR-21 and miR-126 expression and IL-13 concentration was found, showing that these miRNAs are upregulated by IL-13. This cytokine is presented in higher levels in asthmatic patients than in controls due to its involvement in the development of asthmatic inflammation (87).

A downregulated expression of others miRNAs such as miR-18a, miR-27a, miR-128, and miR-155 has been observed in BECs from bronchial brushings from asthmatic subjects. These miRNAs target pathways of inflammation *in silico*, being mothers against decapentaplegic homolog 2 (SMAD2) their common target. When a pool of antagomirs for these miRNAs was used, an increase in the levels of IL-6 and IL-8 was detected (88).

Other groups also employed BECs from healthy controls and asthmatics. Jardim et al. (89) found different expression of miRNAs between asthmatic subjects and those in healthy conditions. One of those deregulated miRNAs was miR-203, which was downregulated in asthmatics. This miRNA targets and regulates the aquaporin gene *AQP4*, which is involved in water transport and osmotic regulation of epithelial lung cells (89).

Studies with epithelial cell lines and murine experimental models have revealed the role of another miRNAs, such as miR-570-3p, which are over-expressed in human epithelial cell lines upon stimulation with TNF-α. miR-570-3p transfection of AECs increases IL-6, chemokine (C-C motif) ligand 5 (CCL5), and CCL4 production, having a synergistic effect with TNF-α administration. The RNA-binding-protein HuR is a target for miR-570-3p, and at the same time, HuR regulates cytokine expression (90), creating a feedback between them.

Another study in AEC lines showed that β-catenin protein, which is involved in cell adhesion, was downregulated by transfection with the miR-3162-3p mimic. This result has been confirmed in an ovalbumin (OVA)-induced mouse asthma model, which showed a higher expression of this miRNA and decreased levels of β-catenin. When anti-miR-3162-3p was administered to these mice, decreasing BALF cell counts and a reduction of airway hyperresponsiveness (AHR) and inflammation, as well as an increase in the β-catenin levels were found (91).

Airway smooth muscle cells are crucial contributors to airflow obstruction through their hypertrophic and hyperplasic processes. Perry et al. (92) observed that the proliferation of ASMCs from severe asthmatic patients and IL-6 release produced by their stimulation with TGF-β1 were mediated through the inhibition of cyclin-dependent kinase inhibitors p21^WAF1^ and p27^kip1^ by miR-221, deregulating the cell cycle. miR-221 is regulated by TGF-β1 and its expression is not altered by corticosteroid treatment. Hu et al. (93) showed that the proliferation of AMSCs is also controlled by miR-10a through suppression of PIK3CA, inhibiting the phosphoinositide 3-kinase (PI3K) pathway, which is involved in cell growth and proliferation. miR-146a and miR-146b were also identified as important miRNAs in asthma pathology, which are highly expressed in stimulated asthmatic ASMCs. They regulate the expression of cyclooxygenase-2, IL-1β, and RNA-binding protein HuR *in vitro*, leading to the regulation of inflammation (94).

### miRNA Expression in Lymphocytes

Expression pattern of miRNA has been studied in other cell populations involved in asthma besides in structural lung cells. In lymphocytes, miR-15a, miR-15b, and miR-20a are downregulated in CD4^+^ T cells from atopic pediatric patients with asthma, compared to atopic and non-atopic controls. Using reporter and immunoprecipitation assays, it was found that vascular endothelial growth factor A (*VEGFA*) is targeted by miR-15a. *VEGFA* is over-expressed in sputum and serum samples of asthmatics and has possible implications in asthma pathogenesis (95). A transcriptomics approach with CD4^+^ and CD8^+^ T cells obtained from severe asthmatics, non-severe asthmatics, and healthy controls showed that CD8^+^ T cells manifested upregulation of the genes is associated with an activated phenotype. qPCR revealed that miR-146a and miR-146b were downregulated in CD4^+^ and CD8^+^ T cells from severe asthmatics compared to non-asthmatic controls, showing that these miRNAs may have a role in CD8^+^ T-cell functions (96).

T helper 2 cytokine production is controlled by phosphatase and tensin homolog, suppressor of cytokine signaling 1 (SOCS1) and deubiquitinase A20. The genes of these proteins are targeted by miR-19a. This miRNA is upregulated in asthmatic Th2 lymphocytes showing a pathophysiological phenomenon occurring in asthma, where those cytokine suppressors are inhibited, and thus inflammation occurs (97).

Additionally, miRNAs from peripheral blood cells are different between asthmatic and healthy subjects. In this way, Dong et al. (98) isolated total RNA from peripheral blood and profiled miRNA expression by microarray and RT-PCR. Their results showed that miR-625-5p, miR-22-3p, and miR-513a-5p were downregulated in peripheral blood samples from asthmatic subjects with dust mite allergy, compared to controls. The targets of these miRNAs (*CBL, PPARGC1B*, and *ESR1*) were inversely upregulated in blood RNA. These genes belong to the PI3K-AKT and nuclear factor κβ (NF-κβ) signaling pathways and may be related to the lower concentrations of IFN-γ, TNF-α, IL-12, and IL-10 in plasma obtained from asthmatics (98).

Expression of miR-155-5p was detected as differentially expressed in allergic-airway-diseased, OVA-induced, steroid-sensitive mice, decreasing its expression when mice were treated with dexamethasone. Specifically, miR-155-5p is more expressed in lung hematopoietic cells. However, when antagomir for miR-155-5p was nasally delivered to mice, no reduction of the disease phenotype or in the expression of target genes was found. This issue was solved by analyzing the anti-miR-155-5p uptake by the different lung cells. Lymphocytes need a higher dose of anti-miR-155-5p than monocytes, macrophages, or neutrophils to develop a phenotype, both *in vitro* as *in vivo* (99).

### miRNA Expression in Body Fluid Samples: Serum, Plasma, Sputum, BALF, and Exhaled Breath Condensate

Asthma is a complex and multifactorial disease influenced by multiple elements. So, not only the cellular component is important in the development of the characteristics that define it but also the soluble inflammatory microenvironment.

Sputum is currently one of the biofluids with increasing applications in asthma research and diagnosis, principally because it is easily harvested and has a direct relationship with the airways status. Maes et al. (100) studied miRNA expression profiles in sputum from healthy, mild-to-moderate asthmatic, and severe asthmatic subjects. They detected an increased expression of miR-629-3p, miR-223-3p, and miR-142-3p in severe asthmatics. These last two miRNAs showed a trend toward increase in neutrophilic asthmatics, showing a positive correlation between their expression and both neutrophil counts and levels of IL-1β, a cytokine involved in inflammatory response. Flow cytometry sorting and *in situ* hybridization showed that miR-223-3p and miR-142-3p were principally expressed by neutrophils and miR-629-3p by epithelial cells, contributing with this load to miRNAs pool from blood torrent, increasing IL-6 and IL-8 levels.

In the search of representative samples derived directly from the airways, exhaled breath condensate was developed as an alternative to sputum. Exhaled breath condensates from healthy, asthmatic, and chronic obstructive pulmonary disease (COPD) subjects were analyzed by qPCR, showing that some miRNAs are downregulated in asthmatics compared to COPD and control individuals (miR-1248, miR-1291, and let-7a); others are downregulated in both asthmatics and COPD groups (miR-328 and miR-21) or downregulated in asthmatic patients than in controls (miR-133a and miR-155). However, all of them share a characteristic: they targeted Th2 mediator genes *in silico* (101). Also, from this type of sample, exosomes have been isolated and their miRNAs content analyzed, showing 11 miRNAs that were differentially expressed between healthy and asthmatic subjects. Exosomes were isolated from these samples, and it was concluded that exosomes contained the majority of the miRNA content (102).

Another sample from lung that has been widely used to analyze inflammatory status in respiratory diseases like asthma is BALF. In this case, the miRNA profile of exosomes from BALF was also studied in healthy and mild intermittent asthmatics, leading to the conclusion that the expression of 16 miRNAs, including members of the let-7 family and miR-203, was able to differentiate asthmatics from healthy subjects at baseline. After air-pollution exposure, 11 of these 16 miRNAs were able to distinguish asthmatic from controls. Expression of these 16 miRNAs also differentiated atopic patients from non-atopic ones, and their target genes belonged to inflammatory response pathways (103).

Previous samples detailed are obtained through invasive methods and/or require trained staff and special equipment and implicate a high time consumption in clinical routine. To overcome this trouble, less invasive methods are currently under development. One strategy may utilize miRNAs obtained from serum or plasma. They are easily isolated and are resistant to RNase degradation due to they usually get around the organism inside exosomes, making them excellent biomarkers (104).

miRNA-21 is one of the most studied miRNAs, being first identified as a biomarker of B-cell lymphoma (105), and subsequently its expression has also been studied in asthma. Elbehidy et al. (106) showed that miR-21 expression is higher in asthmatic patients than in controls and was also higher in steroid-resistant asthmatics compared with steroid-sensitive asthmatics. Moreover, IL-12p35 serum levels were inversely correlated with miR-21 levels, showing a possible role in asthma development through *IL-12p35* repression. Likewise, the ROC curve analysis showed that miR-21 expression levels could be used to differentiate asthmatic from healthy children and to monitor the response to corticosteroid treatment (106). Another group has shown that miR-21 expression was elevated in asthmatics’ serum compared to controls, although they demonstrated that miR-21 expression in serum does not correlate with serum IgE levels (107).

Profiling miRNAs in serum of children with atopic dermatitis and healthy controls by microarray and qPCR showed an upregulation of miR-483-5p and miR-203 in the serum of the dermatitis cases compared to the controls. Analysis by ROC curves demonstrated that serum levels of both miRNAs differentiated healthy from diseased children and that miR-203 was associated with soluble tumor necrosis factor receptor (sTNFR) I and sTNFRII levels in serum, both of which were involved in inflammation (108).

Panganiban et al. (109) showed an upregulation of the expression of miR-1248 and a downregulation of miR-26a, let-7a, and let-7d in serum samples from asthmatics compared to controls, and a negative correlation between miR-26a expression and forced expiratory volume (FEV1) in asthmatics. They confirmed that IL-5 is a target of miR-1248 by using miR-1248 mimics and found that there was bind of this miRNA to the 3′-UTR of IL-5 mRNA (109). This group also described that miR-125b, miR-16, miR-299-5p, miR-126, miR-206, and miR-133b are differentially expressed between asthmatics, allergic rhinitis patients, and controls. Based on these results, they created a random forest model in which the expression of these six miRNAs was able to determine whether a subject was healthy or had disease status with 73 of 79 correct predictions (92.4%; AUC = 9.7). These miRNAs also targeted genes of pathways implicated in immune response and inflammation (110).

An overview of miRNA expression profiling in human samples and their possible roles and function in relation to asthma and allergy is summarized in Tables 1 and 2, respectively.

**Table 1:** MicroRNAs (miRNAs) expression profiling in human samples.

Sample	Differentially expressed miRNAs	Method	Significance	Reference
Airway biopsies	miR-498, miR-155, miR-205 (upregulated), let-7 (downregulated) in allergic rhinitis and asthmatics	Quantitative PCR (qPCR)	Possible disease biomarkers	(83)
	miR-155, let-7 and miR-126 (downregulated), miR-498 (upregulated) in asthmatic	qPCR	Possible disease biomarkers, associated with clinical parameters	(84)
	Studied 227 miRNAs, no differences were found in mild asthmatics	qPCR	Cell-specific miRNA expression	(85)

Epithelial brushings	22 miRNAs found, including downregulation of miR-34/449 families (miR-34c-5p, miR-34b-5p, miR-449a, miR-449b-5p) in asthmatics	Microarray and qPCR	Possible disease biomarkers. Interleukin (IL)-13 represses miR-34/449 family which regulates differentiation of epithelial cells	(86)
	66 miRNAs found, including let-7f, miR-487b, miR-181c (upregulated) and miR-203 (downregulated) in asthmatics	Microarray and qPCR	Possible disease biomarkers. AQP4 gene is targeted by miR-203	(89)
	miR-21 and miR-126 (upregulated) in asthmatics	qPCR	Correlation between miR-21 and miR-126 expression and IL-13 concentration *in vitro*	(87)

Lymphocytes	miR-15a, miR-15b, and miR-20a (downregulated) in asthmatics	qPCR	miR-15a targets vascular endothelial growth factor A in CD4^+^ T cells	(95)
	miR-18a-5p, miR-146a, and miR-146b (downregulated) in asthmatics	Transcriptomics and qPCR	miR-146a and miR-146b may have a role in CD8^+^ cell functions	(96)

Full blood	miR-625-5p, miR-22-3p, and miR–513a-5p (downregulated) in asthmatics	qPCR	miR-625-5p, miR-22-3p, and miR–513a-5p target genes involved in phosphoinositide 3-kinase-AKT and NF-κB signaling pathways	(98)

Sputum	miR-629-3p, miR-223-3p, and miR-142-3p (upregulated) in severe asthmatics	Microarray and qPCR	miR-223-3p and miR-142-3p are expressed by neutrophils. miR-629-3p is expressed by epithelial cells and regulates IL-6 and IL-8	(100)

Exhaled breath condensate	miR-133a and miR-155 (downregulated) in asthmatics	qPCR	Possible disease biomarkers	(101)
	miR-649, miR-1264, miR-2861, miR-574-5p (upregulated) and miR-453, miR-4256, miR-556-5p (downregulated) in asthmatic	miRNome and qPCR	Possible disease biomarkers	(102)

Bronchoalveolar lavage fluid exosomes	Let-7a, miRNA-21, miRNA-24, miR-26a, miRNA-99a, miRNA-200c (downregulated), and miRNA-658, miRNA-1268 (upregulated) in asthmatics	Microarray and qPCR	Possible disease biomarkers	(103)

Plasma	miR-125b (downregulated) and miR-16, miR-299-5p, miR-126, miR-206, miR-133b (upregulated) in asthmatics	Microarray and qPCR	Possible disease biomarkers	(110)

Serum	miR-21 (upregulated) in asthmatic compared to controls and upregulated in steroid-resistant asthmatics compared with steroid-sensitive asthmatics	qPCR	Possible disease biomarker. miR-21 targets IL-12p35	(106)
	miR-21 upregulated in asthmatics	qPCR	Possible disease biomarker. miR-21 does not correlate with serum IgE levels	(107)
	miR-483-5p and miR-203 upregulated in atopic dermatitis compared to controls	Microarray and qPCR	Possible disease biomarkers. miR-203 is associated with soluble tumor necrosis factor receptor (sTNFRI) and sTNFRII levels	(108)
	miR-1248 (upregulated), miR-26a, let-7a, and let-7d (downregulated) in asthmatics	qPCR	Possible disease biomarkers. IL-5 is target of miR-1248. Correlation between miR-26a and FEV1%	(109)

**Table 2:** MicroRNAs (miRNAs) function in asthma.

Study model	miRNA	Gene target	Significance	Reference
Airway smooth muscle cells (ASMCs)	miR-221	p21^WAF1^ and p27^kip1^	Regulation of cell proliferation	(92)
ASMCs	miR-10a	PIK3CA	Regulation of cell growth and proliferation	(93)

ASMCs	miR-146a and miR-146b	Cyclooxygenase-2, IL-1β, HuR	Regulation of cytokine expression and inflammation	(94)
Airway epithelial cells (AECs)	miR-570-3p	HuR	Regulation of cytokine expression	(90)
AECs/ovalbumin (OVA)-induced asthma mouse model	miR-3162-3p	β-catenin	Regulation of cell adhesion, and inflammation	(91)

Bronchial epithelial cells (BECs)	miR-18a, miR-27a, miR-128 and miR-155	SMAD2	Regulation of cytokine expression	(88)
BECs	miR-203	AQP4 (non-validated)	Osmotic regulation	(89)

BECs	miR-449	NOTCH1	Airway mucous metaplasia	(86)
BECs	miR-629-3p	IL-6 and IL-8 (non-validated)	Regulation of cytokine expression	(100)
OVA-induced asthma mouse model	miR-155-5p	ACVR2A, TAB 2 (non-validated)	Anti-miR-155-5p uptake depends on cell type	(99)
Th2 lymphocytes/OVA-induced asthma mouse model	miR-19a	Phosphatase and tensin homolog, SOCS1, A20	Regulation of cytokine expression and inflammation	(97)

OVA-induced asthma mouse model	miR-126	PU.1, target of Myb protein 1	Regulation of inflammation	(130, 131)

OVA-induced asthma mouse model	anti-miR-221		Regulation of inflammation	(132)
CD4^+^ T cells/OVA-induced asthma mouse model	Let-7	IL-13	Regulation of cytokine expression and inflammation	(133, 134)

CD4^+^ T cells	miR-15a	Vascular endothelial growth factor A	Regulation of angiogenesis, cell migration	(95)

Jurkat T cells/peripheral blood mononuclear cells	miR-1248	IL-5	Regulation of inflammation	(109)
Blood total RNA	miR-625-5p, miR-22-3p, miR–513a-5p	CBL, PPARGC1B, and ESR1 (non-validated)	Regulation of cytokine expression	(98)
Serum RNA	miR-21	IL-12p35 (non-validated)	Regulation of cytokine expression	(106)

Serum RNA	miR-203	Soluble tumor necrosis factor receptor (sTNFRI) and sTNFRII (non-validated)	Regulation of cytokine expression	(108)

## Exosomes and miRNAs: Biomarkers and Potential Therapeutic Tools

The recent knowledge of exosomes and miRNA structure and function has allowed that both can be used as molecular biomarkers for diagnostic and prognostic purposes and raised the possibility of developing potential therapeutic tools (111). Detection of miRNAs in human body fluids (including serum, plasma, urine, and saliva) has led to studies focused on the development of miRNA tests as disease biomarkers. Moreover, these non-coding RNAs sequences are usually transported in microparticles and exosomes, which prevents their degradation, and this bestows an advantage to utilize them for these studies (112). In particular, cancer research is a field in which miRNAs and exosomes have become relevant, for use as biomarkers as well as therapeutic tools (113–120). In addition, there is an increased interest in different pathologies (121–127), including pulmonary diseases (128, 129).

So, asthma is one of the medicine field in which modulation of miRNA expression as a therapeutic approach is being explored. Inhibition of miR-126 has been tried in mouse models of asthma, showing that regulation of this miRNA targets toll-like receptor 4 (TLR4) through upregulation of transcription factor PU.1 and upregulation of target of Myb protein 1, blocking IL-1β and TNF-α and thus inhibiting the asthmatic inflammation phenotype (130, 131). Another group confirmed that the modulation of miRNA expression can be a potential treatment. OVA-induced mice were treated with an anti-miR-221, causing a reduction in cell infiltration and eosinophils in BALF (132).

Let-7 is one of the most studied miRNAs in asthma. Kumar et al. (133) showed a positive binding of let-7 to IL-13 3′-UTR mRNA by luciferase reporter assays *in vitro*. Also, when T cells from human PBMCs were cultured under T-polarizing conditions and transfected with let-7 family miRNAs, a reduction in IL-13 production was observed. *In vivo*, they found less expression of let-7 in OVA-induced asthma mice model, and when let-7 mimic was delivered into these mice, a reduction of IL-13 and other asthma features such as cell infiltration and AHR were observed (133). Polikepahad et al. (134) also described the let-7 family as strongly expressed in lungs, independently of allergen exposure. When they analyzed IL-13 mRNA and let-7 expression in naive CD4^+^ T cells, IL-13 was upregulated and let-7 was downregulated. Administration of anti-let-7 before OVA challenge in OVA mice models decreased IL-13 levels, and they observed a reduced AHR, cell infiltration, and mucus production (134).

These results demonstrate that more research is needed to elucidate miRNA modulation as a therapeutic target, although initial studies show promising potential. Moreover, another field of research is focused on interventions over exosomes. So, diverse agents could act directly or indirectly on exosome biogenesis, secretion, and function. This exosome modulation opens a new way in future therapeutic approach to control different mechanisms implicated in asthma development (69).

## Conclusion

Exosomes and miRNAs are the most important areas of biomedical research emerging in last years. Both components of cellular machinery are rising in all fields of medicine as powerful potential tools with relevant roles in prophylactic, diagnostic, and possible new therapeutic approaches. The potential use of exosomes and miRNAs is being demonstrated in multiple pathologies. In respiratory field, specifically asthma pathology, exosomes and miRNAs from several cell types contribute, in a different manner depending of context, to worsen or improve symptoms of pathology. Their capacities to get around other areas convert them in an exceptional potential tool to modulate pathologic processes. As we have previously detailed, both structural lung cells and effector cells contribute to exosomes pool and miRNAs content that characterize the inflammatory microenvironment that determine the clinical symptoms and severity of asthma and allergic disease.

However, there are still many unknowns that need to be solved. Which are the elements that specifically modulate each miRNA in each context? Which stimulus affect to content of exosomes? Could we regulate the quantity and composition of these exosomes? So, exosomes and miRNAs could be new and crucial elements in effort of traslacional research to establish a “signature” that allows a better diagnostic, prophylactic, or therapeutic approach, although a better understanding of the impact and functional mechanisms of these elements is necessary.

## Author Contributions

VP conceived the manuscript and JC, BS, and JR-M contributed to the writing of the manuscript and all approved its final content.

## Conflict of Interest Statement

The authors declare that the research was conducted in the absence of any commercial or financial relationships that could be construed as a potential conflict of interest.

## Abbreviations

AECs, airway epithelial cells; AHR, airway hyperresponsiveness; APCs, antigen-presenting cells; ASMCs, airway smooth muscle cells; BALF, bronchoalveolar lavage fluid; BECs, bronchial epithelial cells; COPD, chronic obstructive pulmonary disease; COX, cyclooxygenase; DCs, dendritic cells; ECP, eosinophil cationic protein; EDN, eosinophil-derived neurotoxin; EPO, eosinophil peroxidase; HSP, heat shock protein; ICAM, intercellular adhesion molecule; IFN-γ, interferon-gamma; LFA, lymphocyte function-associated antigen; LTs, leukotrienes; MBP, major basic protein; MCs, mast cells; MDM, monocyte-derived macrophages; MHC, major histocompatibility complex; miRNAs, microRNAs; MVBs, multivesicular bodies; PBMCs, peripheral blood mononuclear cells; qPCR, quantitative PCR; shRNAs, short hairpin RNAs; siRNAs, small interference RNAs; sTNFR, soluble tumor necrosis factor receptor; Th, T helper; TNF-α, tumor necrosis factor alpha; VEGFA, vascular endothelial growth factor A.
